# Filamentous fungal applications in biotechnology: a combined bibliometric and patentometric assessment

**DOI:** 10.1186/s40694-021-00131-6

**Published:** 2021-12-28

**Authors:** Pamina Füting, Lars Barthel, Timothy C. Cairns, Heiko Briesen, Stefan Schmideder

**Affiliations:** 1 Chair of Process Systems Engineering, School of Life Sciences Weihenstephan, Technical University of Munich, 85354 Freising, Germany; 2 Chair of Applied and Molecular Microbiology, Faculty III Process Sciences, Institute of Biotechnology, Technische Universität Berlin, Berlin, Germany

**Keywords:** Filamentous fungi, Citric acid, Organic acid, Enzyme, Glucoamylase, *Aspergillus*, *Trichoderma*, Penicillin, Wastewater, Biofuel, Intellectual property

## Abstract

**Background:**

Processes and products employing filamentous fungi are increasing contributors to biotechnology. These organisms are used as cell factories for the synthesis of platform chemicals, enzymes, acids, foodstuffs and therapeutics. More recent applications include processing biomass into construction or textile materials. These exciting advances raise several interrelated questions regarding the contributions of filamentous fungi to biotechnology. For example, are advances in this discipline a major contributor compared to other organisms, e.g. plants or bacteria? From a geographical perspective, where is this work conducted? Which species are predominantly used? How do biotech companies actually use these organisms?

**Results:**

To glean a snapshot of the state of the discipline, literature (bibliometry) and patent (patentometry) outputs of filamentous fungal applications and the related fields were quantitatively surveyed. How these outputs vary across fungal species, industrial application(s), geographical locations and biotechnological companies were analysed. Results identified (i) fungi as crucial drivers for publications and patents in biotechnology, (ii) enzyme and organic acid production as the main applications, (iii) *Aspergillus* as the most commonly used genus by biotechnologists, (iv) China, the United States, Brazil, and Europe as the leaders in filamentous fungal science, and (v) the key players in industrial biotechnology.

**Conclusions:**

This study generated a summary of the status of filamentous fungal applications in biotechnology. Both bibliometric and patentometric data have identified several key trends, breakthroughs and challenges faced by the fungal research community. The analysis suggests that the future is bright for filamentous fungal research worldwide.

**Supplementary Information:**

The online version contains supplementary material available at 10.1186/s40694-021-00131-6.

## Background

There are an estimated 2 to 5 million fungal species on Earth [1, 2]. Unicellular yeasts replicate by budding and are distinguished from filamentous fungi that colonise substrates by the growth of multicellular, highly polar cells termed hyphae [3]. The success of the filamentous lifestyle is evidenced by the near-ubiquity of these organisms found in virtually all aquatic and terrestrial environments. The life modes of filamentous fungi are extremely diverse and include pathogens of animals, plants and other organisms and, alternatively, mutualists, parasites, symbionts, and free-living microbes [4].

Filamentous fungi are heterotrophs and have evolved remarkably diverse nutritional capabilities, including growth on complex lipids, proteins and polysaccharides [5]. This robust heterotrophy has led to the century-long application of filamentous fungi as microbial cell factories for two reasons [6]. First, it enables the use of cheap, readily available waste sources as feedstocks. Second, growth on complex substrates requires the secretion of hydrolytic enzymes that themselves are highly prized molecules for biotechnological use. When combined with the propensity to secrete various industrially used organic acids and bioactive secondary metabolites, including but not limited to beta-lactam antibiotics, filamentous fungi are powerful components of the now emerged and expanding biotechnological revolution [7, 8]. Several molecules from this growing product portfolio [e.g. citric acid (used as a flavouring agent, cleaning product and platform chemical), the enzyme glucoamylase (used to breakdown starch in the food industry) and statins (used to reduce cholesterol in humans)] now constitute growing, multimillion-dollar industries each year [3].

However, applications of filamentous fungi are not limited to the fermentation, isolation and use of their secreted enzymes, acids or secondary metabolites. Technological developments are as varied as the ecological niches colonised by these organisms and include the development of myco-leather and other textiles, building materials, biosensors for disease, wastewater treatment, sustainable meat substitutes, amongst many other applications [7, 9–11]. Indeed, the future replacement of the current petroleum-based economy with a sustainable bioeconomy may rely significantly on applied filamentous fungal science [7–9].

Given these advances in the past two decades, it is now time to quantitatively assess the status of the field. Some studies have addressed the literature output of a single species (e.g. *Aspergillus niger* [6]) or qualitatively reviewed patent outputs with regards to specific subdisciplines (e.g. bio-based materials [9]) and from interdisciplinary perspectives [11]. While literature (bibliometry) and patent (patentometry) outputs are powerful analytical approaches in their own right, combining these techniques in a single study can provide a complementary and holistic understanding of fundamental research and its translation to applied science over a specific period [12]. Moreover, cataloguing such outputs and studying how they vary across fungal species, industrial application(s), geographical locations, and biotech companies enable the delineation of the key trends, breakthroughs and challenges faced by fungal research. This study mined publicly available repositories for literature and patent outputs derived from filamentous fungal science and identified five key trends from these datasets, which are timely summaries of the status of these organisms in biotechnological applications.

## Results

### Fungi are crucial drivers for literature and patent outputs in biotechnology

**Fig. 1:**
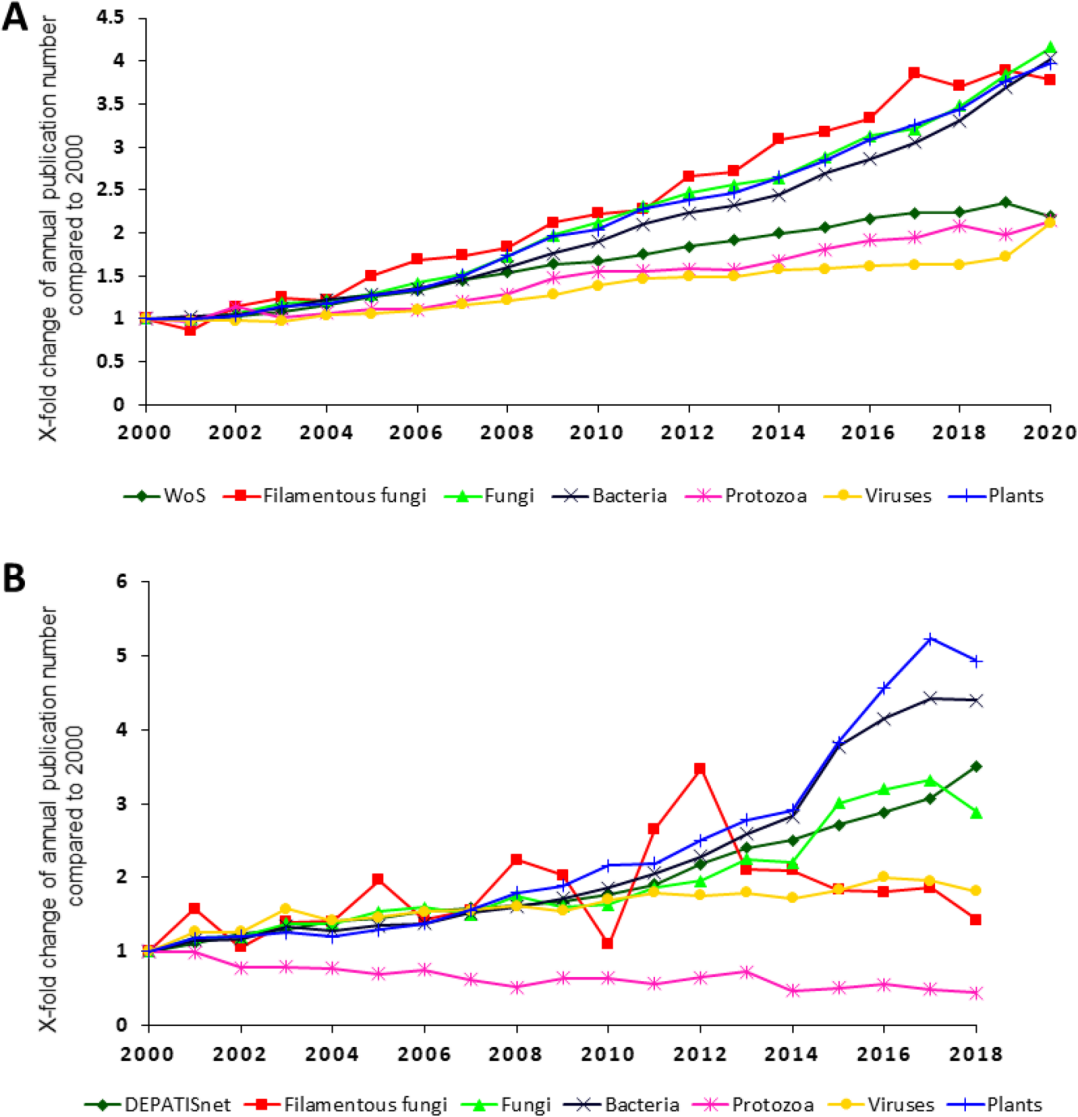
Annual publication and patent frequencies amongst selected groups. The annual publication number was divided by the publication number in 2000. **A** Literature. Data for WoS refer to all publications in Web of Science. **B** Patents. Data for DEPATISnet refer to all patents published in DEPATISnet. “Filamentous fungi” are a subgroup of “fungi” and the group “fungi” includes publications/patents about filamentous fungi

To estimate the relative importance of filamentous fungi in biotechnological applications, literature and patent outputs amongst six key cohorts were analysed and compared (Fig. 1; Table 1). Data were collected from Web of Science (WoS) and DEPATISnet from 2000 to 2020 and from 2000 to 2018, respectively. As explained in the Methods Section, the different time periods are caused by the time lag (approximately 18 months) patents get filled and published.

**Table 1:** Annual number of publications of different cohorts in biotechnology and all publications in Web of Science (WoS; literature) and DEPATISnet (patents)

	Year	Total	Filamentous fungi	Fungi	Bacteria	Protozoa	Viruses	Plants
WoS	2000	1,243,835	116	2211	6738	252	4651	10,226
DEPATISnet	2000	1,738,308	76	3314	8274	360	6542	19,805
WoS	2020	2,725,090	439	9204	27,179	539	9837	40,673
DEPATISnet	2018	6,091,925	108	9560	36,407	156	11,827	97,628

In Fig. 1, the annual publication number was divided by the publication number in 2000. Figure 1A indicates the rapid expansion of the biotechnology literature compared to scientific output as a whole in the last 20 years. Notably, research on filamentous fungi grew comparably to plants and bacteria, the latter two organisms containing well-established cell factories to produce an extremely diverse and valuable product portfolio, including vaccines, medicines, food, platform chemicals, industrial enzymes and many other molecules. This analysis also revealed that research utilising protozoa and viruses grew less in literature outputs than other cohorts, an observation that further highlights the relative importance of filamentous fungi in biotechnological research.

The development of patents covering biotechnological applications of the same cohorts showed the biggest increase in growth for the utilisation of plants and bacteria, clearly outperforming patent output as a whole (Fig. 1B). Filamentous fungi and viruses demonstrated a smaller increase in annual growth, which exceeded protozoa whose annual patents have declined.

The total numbers of annual publications and patents of the given cohorts in Table 1 again demonstrate the outstanding importance of plants and bacteria for biotechnological applications. In addition, fungi and viruses appeared to be already widely applied in both research and industry. Although the utilisation of filamentous fungi for both purposes showed an increase in the displayed time, the total number of publications and patents dealing with this group of organisms was small compared to other cohorts such as bacteria and plants.

### Enzyme and organic acid production are the main outputs for literature and patents in the last 20 years

**Fig. 2:**
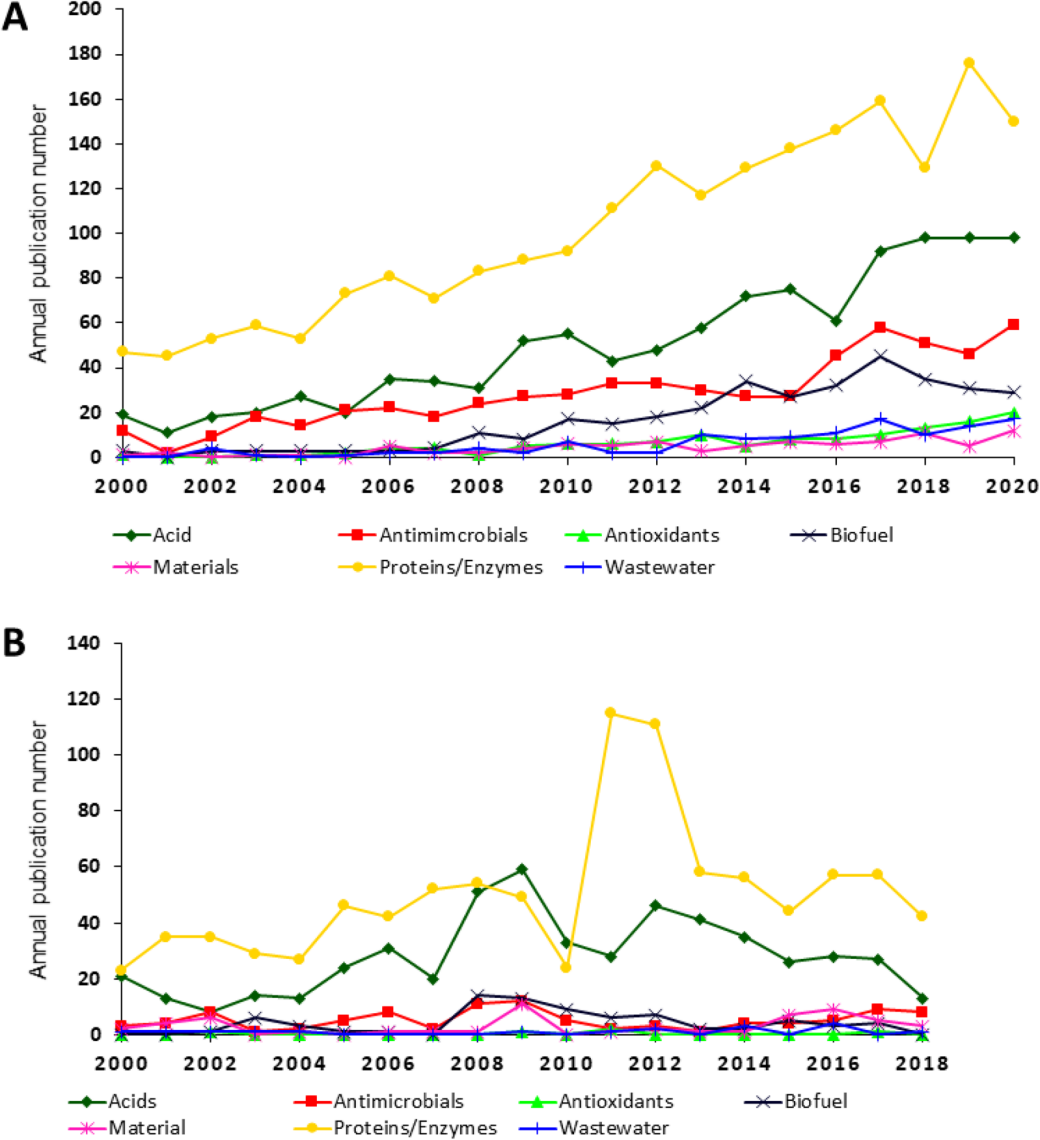
Annual publication number concerning filamentous fungal products and/or applications. **A** Literature and **B** patents. Based on all literature and patents on filamentous fungi, 62% and 54% are covered by at least one product/application, respectively

The product repertoire of filamentous fungi rapidly expanded in the last decade and included numerous proteins, acids, secondary metabolites and biomass, all of which can be used in diverse applications, including industrial microbiology, wastewater treatment (filamentous fungi are predominantly used to degrade organic compounds [13]), construction, and many others [3]. To identify the most important products and/or applications, literature and patent outputs were assigned into various categories (Fig. 2).

This analysis indicated that protein production, especially enzyme production was by far the most abundant application of filamentous fungi in research in the last 20 years, followed by acid production (Fig. 2A). Research on antimicrobials, biofuels, antioxidants, wastewater treatment and biomaterials seemed less intense, although publications regarding biofuel and antimicrobial production increased from 2000 to 2020 (Fig. 2A). The prominent utilisation of filamentous fungi for protein/enzyme and acid production was also clearly discernable in the quantification of patents (Fig. 2B). These data were consistent with historical applications of these organisms, including organic acid production for > 100 years and the production of industrially utilised proteins since the 1960s (e.g. citric acid and glucoamylases in *A. niger*, respectively) [6]. Taken together, assigning literature and patent outputs into six exemplar categories confirmed that despite the emergence of new products and applications, the major use for filamentous fungi in biotechnology remained protein, enzyme and acid production.

### Aspergillus is the key genus for biotechnologists

**Fig. 3:**
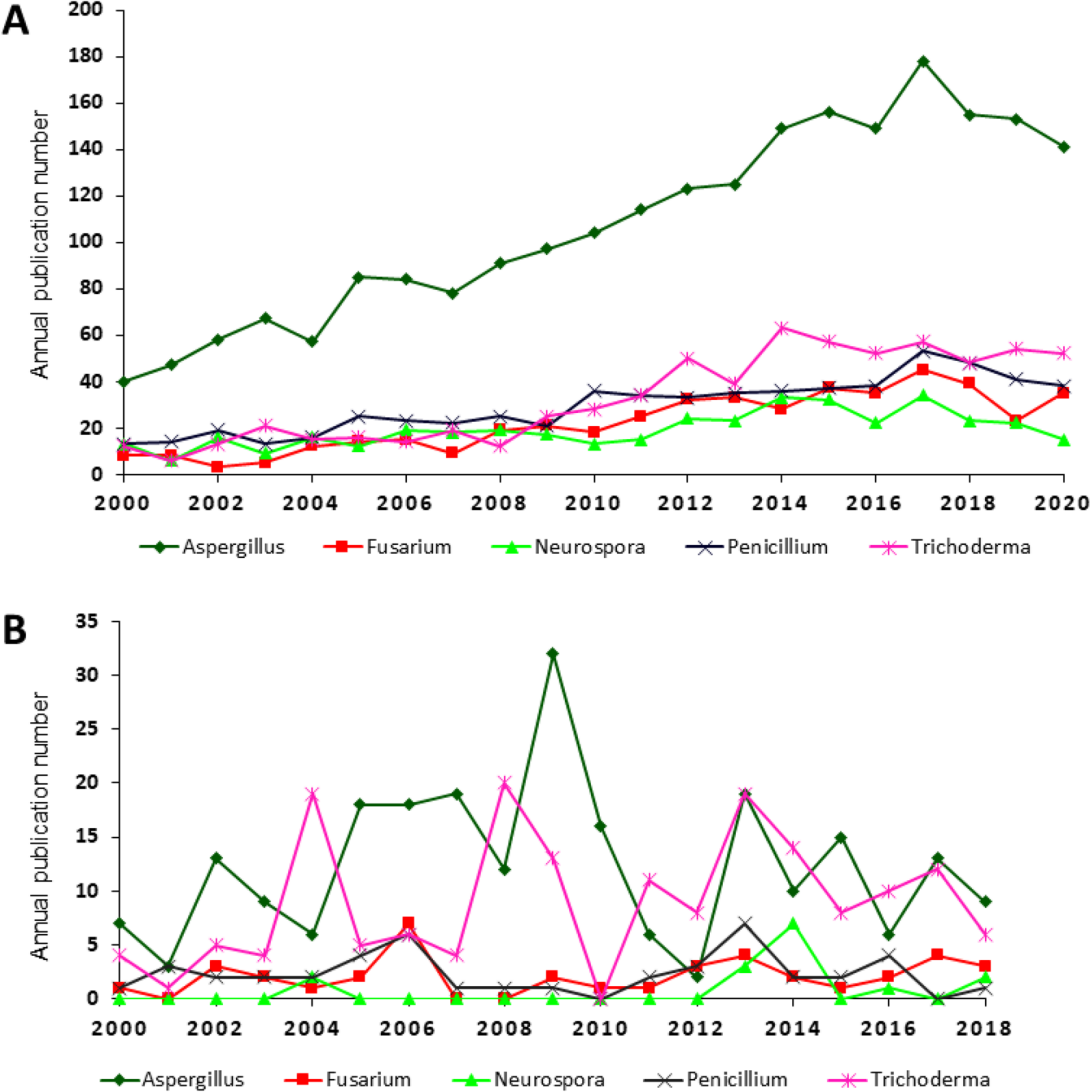
Annual number of publications from filamentous fungal genera Aspergillus, Fusarium, Neurospora, Penicillium and Trichoderma. **A** Literature and **B** patents. Based on all literature and patents on filamentous fungi, 59% and 16% are covered by at least one genera, respectively

To determine the main genera harnessed in biotechnological applications, literature and patent outputs were assigned to *Aspergillus* spp., *Neurospora* spp., *Fusarium* spp., *Penicillium* spp. and *Trichoderma* spp. (Fig. 3).

Figure 3A clearly demonstrates that *Aspergillus* spp. are the most commonly utilised species in biotechnological applications. There were approximately three times as many publications derived from *Aspergillus* spp.-related research compared to the second most abundant genus, *Trichoderma* spp. The trend in patents was comparable (Fig. 3B). Also, *Penicillium* spp., *Fusarium* spp. and *Neurospora* spp. were the least abundant of the five investigated genera. *Trichoderma* spp. was used comparably often as *Aspergillus* spp., especially in the last decade.

**Fig. 4:**
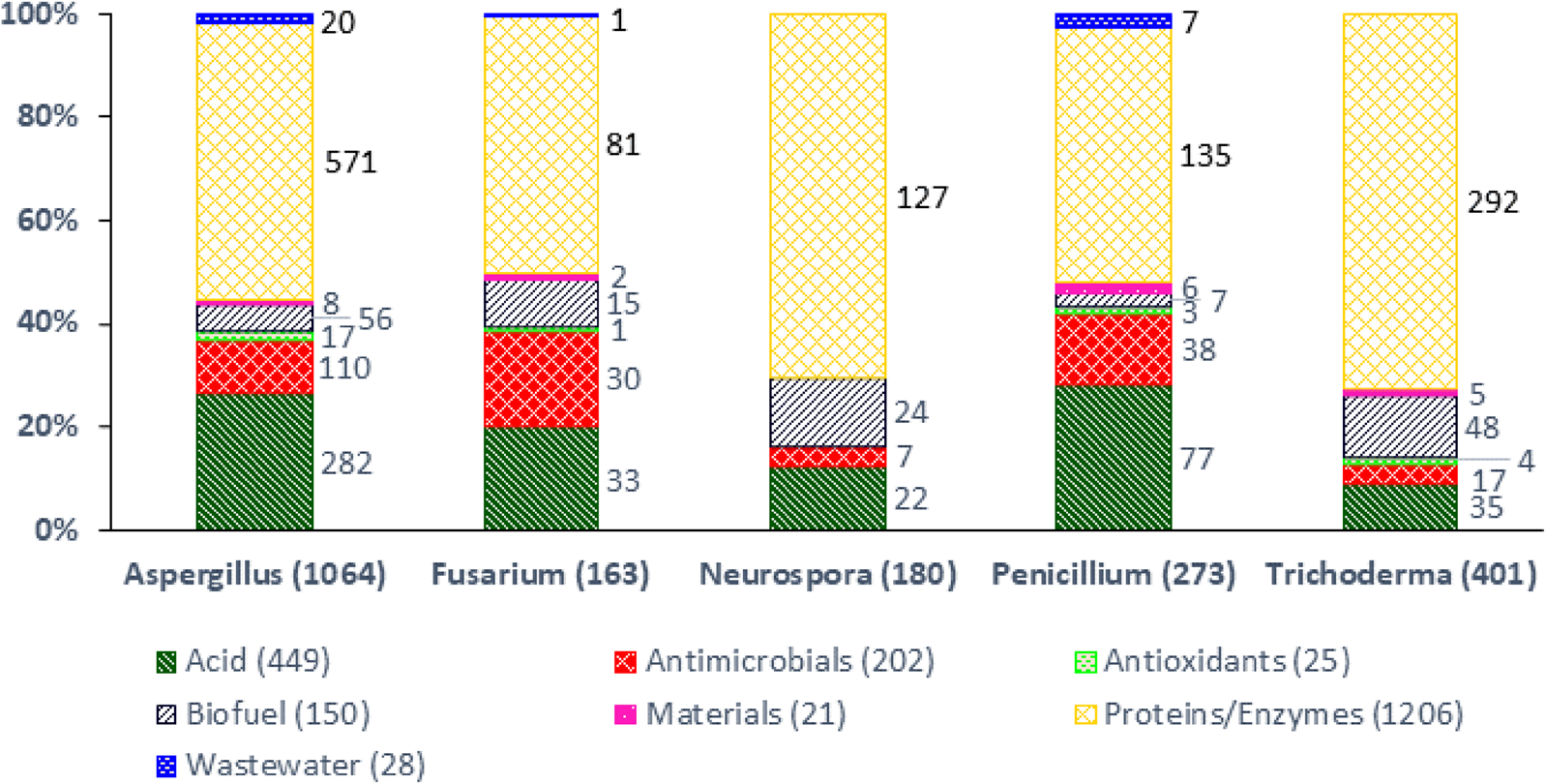
Products/applications of filamentous fungal genera *Aspergillus*, *Fusarium*, *Neurospora*, *Penicillium* and *Trichoderma* in the literature from 2000 to 2020

To determine how the five filamentous fungal genera are utilised for different applications or products, we combined data depicted in Figs. 2A and 3A into a single dataset (Fig. 4). This analysis was limited to research publications, because the genus applied in a certain patent is often not mentioned in the abstract or title. More precise, 59% (research publications) and 16% (patents) were covered by at least one of the mentioned five genera.

This approach confirmed that irrespective of the genera used by researchers, most applications of filamentous fungi were related to protein and enzyme production (Fig. 4). Given the role of *A. niger* as a production host for citric and gluconic acid and *Aspergillus terreus* for producing itaconic acid [3], *Aspergillus* was unsurprisingly the most commonly used genus for the study of organic acids. Interestingly, when the output for each genus was viewed as a percentage of the respective total, *Penicillium* and *Fusarium* spp. were more commonly employed for the study of antimicrobials. Clearly, *Penicillium* spp. were utilised for the study and production of beta-lactam antibiotics. Data supported the emergence of *Fusarium* spp. as promising natural product reservoirs for antimicrobials, including enniatins, antibiotic Y, aurofusarin, beauvericin and others [14]. From the perspective of biofuels, it is clear that the main genera were *Aspergillus*, *Neurospora* and *Trichoderma*. Again, when viewing as a percentage of the total output, *Trichoderma* spp. were often employed for biofuel research, an observation consistent with their high production of carbohydrate-activating enzymes (CAZys), including cellulases and hemicellulases, necessary to degrade plant waste for biofuel production [15]. The latter observation also demonstrated a possible limitation to this analysis: enzyme production for utilisation in biofuel processing may count ambiguously or into two categories during data analysis (see Discussion). In general, more than half of the articles analysed in Fig. 4 were related to *Aspergillus* spp., reinforcing the importance of *Aspergillus* spp. in modern biotechnology.

### People’s Republic of China, the United States (USA), Brazil and Europe are the leaders in filamentous fungal science

**Fig. 5:**
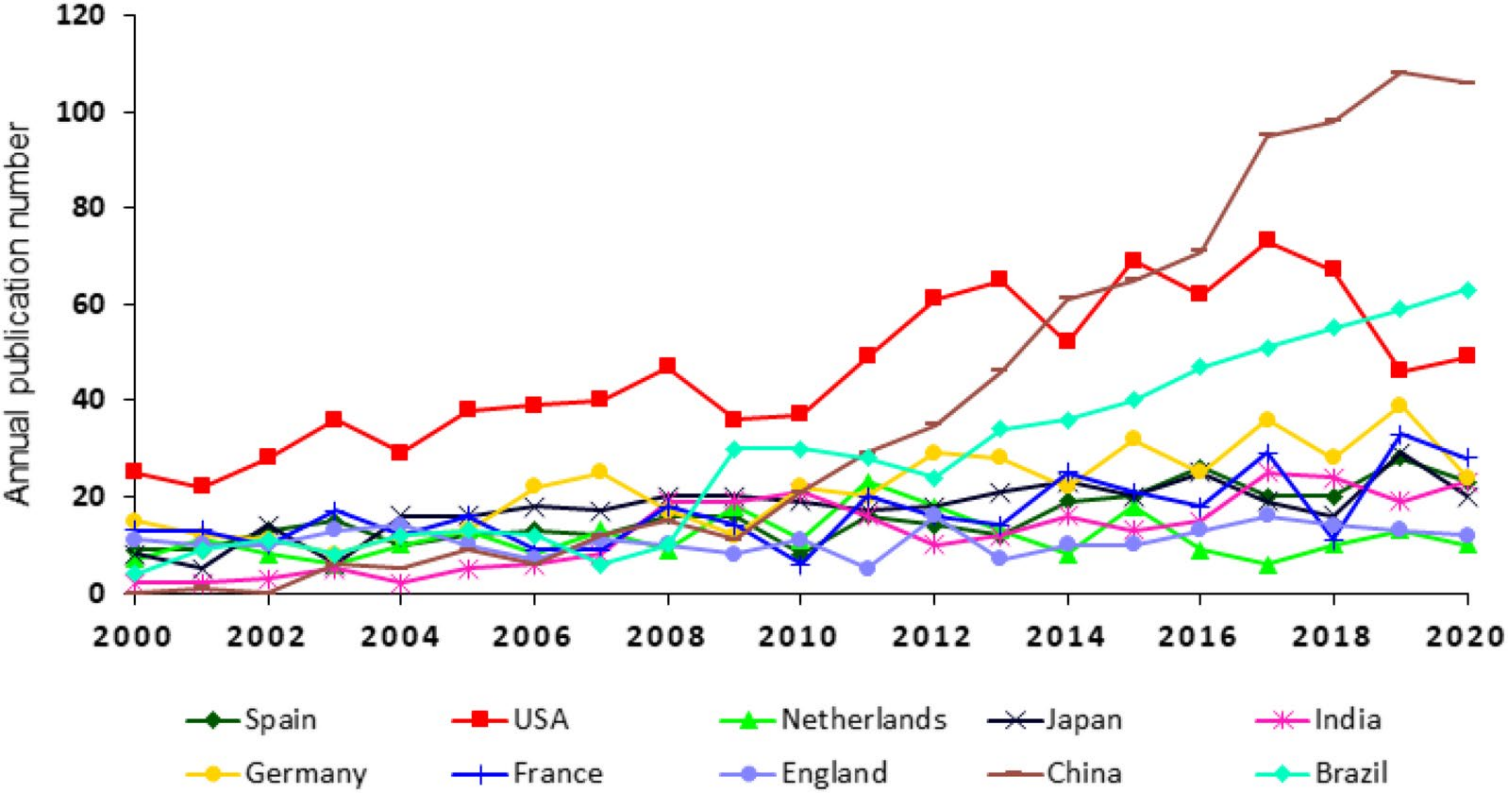
Annual number of published literature dealing with the application of filamentous fungi. The nationality is based on the corresponding author’s department. The 10 countries with the most publications from 2000 to 2020 are shown

Estimating filamentous fungal research outputs from individual nations could be useful for the research community, for example, to aid Ph.D. students/postdoctoral researchers when selecting where to study and/or train or to facilitate collaborative projects between established and/or emerging countries in the field. Figure 5 shows the 10 countries with the most publications on the use of filamentous fungi in the last 20 years. The authors of these countries were responsible for 70% of all research papers analysed in this study.

Three dominant countries could be recognised. From the beginning of the investigation until 2013, the USA published significantly more manuscripts in this field than any other country, followed by a decrease in publication numbers in 2019 and 2020. People’s Republic of China was a comparably small player until 2009, after which a notable annual increase in the number of publications was observed, allowing People’s Republic of China to overtake the output of the USA since 2014. In 2020, China had more than double as many published manuscripts in the field of filamentous fungi used in biotechnological applications than the USA. The third up-and-coming big player was Brazil. Similar to People’s Republic of China, Brazil did not show a significantly high number of publications until 2008 but since then showed a strong annual increase in publication number. Five European countries, Spain, Germany, France, The Netherlands and England, were represented, showing the high impact of this continent.

**Fig. 6:**
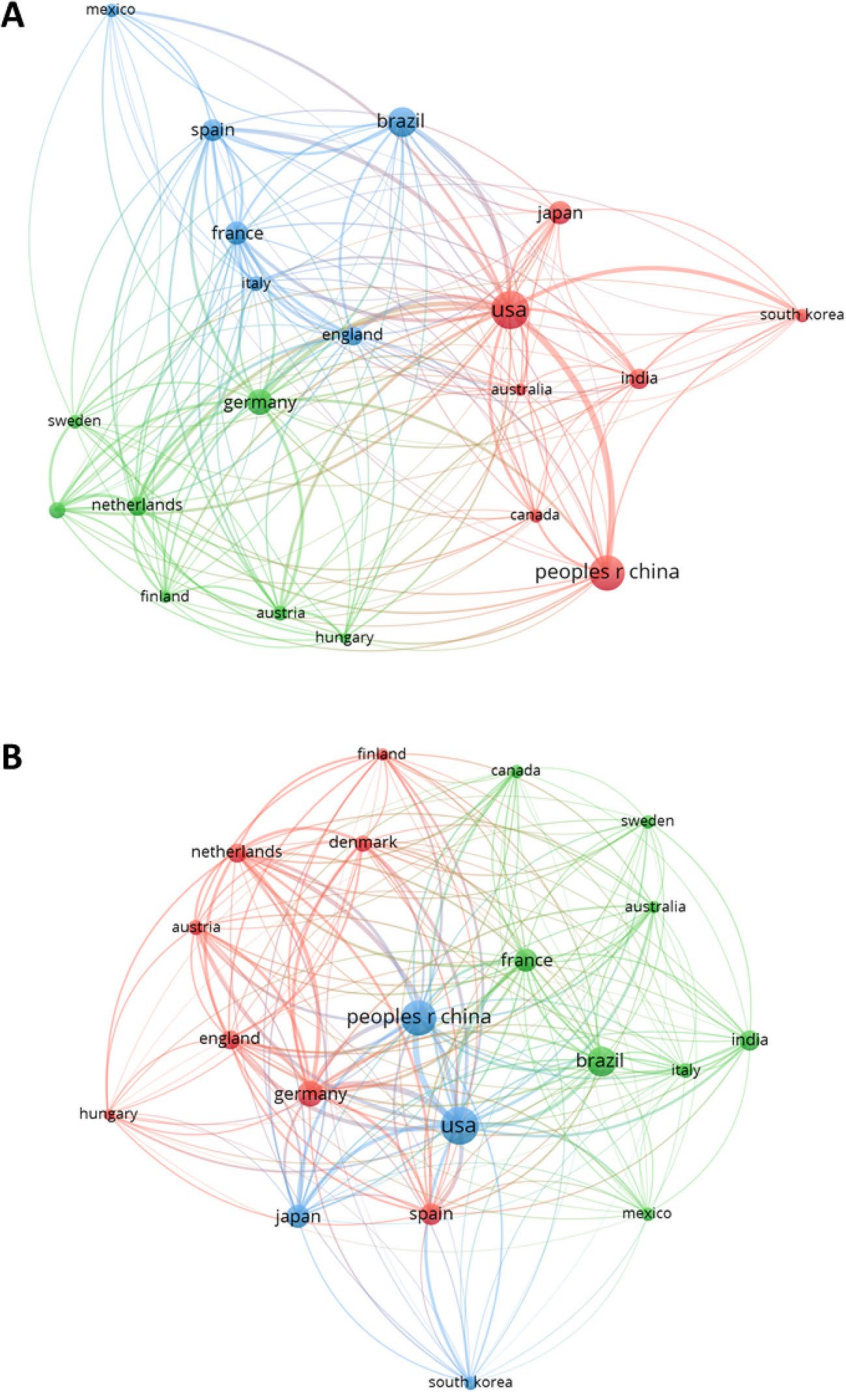
National networks of published literature dealing with the application of filamentous fungi from 2000 to 2020. **A** Coauthorship network and **B** citation network. The nationality was based on the corresponding author’s department. The 20 countries with the most publications from 2000 to 2020 are shown

To define and investigate the international network dealing with filamentous fungi in biotechnological applications, this study analysed the connections amongst the 20 most abundant nationalities of researchers’ departments, coauthoring the same manuscripts, as well as the nationalities of the corresponding authors’ departments, citing each other’s manuscripts (Fig. 6). Clustering and visualisation were performed with VOSviewer [16].

The coauthorship network (Fig. 6A) unveiled three different main clusters that seemed to be at least partly geographically organised. The green cluster consisted of northern or central European countries, including Germany, Sweden, The Netherlands, Finland, Austria and Hungary. The blue cluster combined several southern European countries, such as Spain, France, and Italy, and others that did not belong into this geographical group, including England, Brazil and Mexico. The red cluster was a very international group of countries not located next to each other, including the USA, People’s Republic of China, Japan, India, Australia, Canada and South Korea. Besides these clusters, collaborative links between all present countries could be observed. Thus, this analysis indicated three large collaborative networks in filamentous fungal science, which were generally discernable from coauthor relationships. Nevertheless, the community was not fixed into collaborative subdivisions, as evidenced by coauthored manuscripts between authors within these three networks.

The national citation network (Fig. 6B) differed from the coauthorship network. Again, three main clusters could be observed. However, in general, most of the analysed countries were located closer to each other on the network map, showing strong interlacing when it comes to international citations. The red cluster consisted of a total of eight European countries, mostly in northern and middle Europe. The green cluster was very international, including European (France, Sweden and Italy) as well as American (Canada, Brazil and Mexico) and Asian (India) countries. The blue cluster consisted of People’s Republic of China, the USA, Japan and South Korea. Notably, the five countries that played a major role in both networks were People’s Republic of China, the USA, Brazil, Germany and France.

Table 2 ranks the literature output of the 10 countries with the highest number of publications in the field of filamentous fungi. An additional indicator was the total number of publications per country divided by the respective population of 2020. This allowed for a weighting by the population. Further, different bibliometric indices were used to rank the output of each country. More precise, the H-index [17], g-index [18], and i10-index (Google Scholar) were analysed. In this analysis, the H-index quantifies the number of publications with at least the same amount of citations. For example, 51 of the 802 manuscripts published by Chinese corresponding authors were cited at least 51 times. The g-index is the number of the top g articles that received together at least g^2^ citations. For example, the top 74 Chinese manuscripts received at least 74^2^ = 5476 citations. The i10-index shows the number of publications with at least 10 citations.

**Table 2:** Number of publications and bibliometric indices of countries considering published literature dealing with the application of filamentous fungi from 2000 to 2020

Country	Publications	Country	Publications per 100,000 inhabitants	Country	H-Index	Country	g-Index	Country	%	i10-Index
USA	970	Netherlands	141	USA	101	USA	175	Netherlands	79	192
China	802	Spain	71	Germany	72	Germany	140	USA	73	709
Brazil	584	Germany	54	Netherlands	58	France	117	Germany	67	303
Germany	453	France	54	France	57	England	115	France	67	235
Japan	367	England	34	Japan	53	Spain	107	England	65	151
France	352	USA	29	Brazil	52	Netherlands	106	Spain	64	213
Spain	331	Japan	29	China	51	Brazil	105	Japan	62	228
India	267	Brazil	27	England	50	Japan	103	India	48	128
Netherlands	242	China	6	Spain	50	India	76	China	47	379
England	231	India	2	India	41	China	74	Brazil	47	273

The nationality was based on the corresponding author’s department. The 10 countries with the most publications are shown. To calculate the number of publications per 100,000 inhabitants, the number of publications was divided by the respective population of 2020, which was taken from a report of the United Nations (UN) (https://population.un.org/wpp/Download/Standard/Population/). Contrary to bibliometrics, the UN only considered the United Kingdom (UK). Thus, the population of England in the year 2020 was taken from UK's Office for National Statistics (https://www.ons.gov.uk/peoplepopulationandcommunity/populationandmigration/populationestimates/bulletins/annualmidyearpopulationestimates/mid2020).

Consistent with data in Fig. 5, the total number of publications dealing with filamentous fungi in biotechnological applications is the highest for the USA, People’s Republic of China and Brazil, with 970, 802 and 584 in the last 20 years, respectively. However, weighting the total number of publications with the population of each country, this ranking looks different. In this analysis, The Netherlands is in first place, followed by Spain, Germany, France, and England. USA, China and Brazil are ranked sixth, eighth and ninth, respectively. The USA also achieved the highest rank in the list of H- and g-indices and was second when comparing the i10-index. People’s Republic of China and Brazil were only placed in the lower half of each index list regardless of their high total number of publications. One reason for this phenomenon might be the recent increase in the total publications for People’s Republic of China and Brazil, which reduced the amount of time for these papers to be cited. Germany, ranking fourth in terms of total publication number, was second in the list of H- and g-indices and third in the i10-index list. Remarkably, The Netherlands, only ranking ninth in the total publication number list, reached first place in the population weighted list, third place for the H-index and first place for the i10-index, demonstrating a high international impact.

### Key players in industrial fungal biotechnology

Finally, this study wanted to determine which major biotechnology companies utilised filamentous fungi and why. The distribution of the exploitation of filamentous fungi for biotechnological applications in the modern industry can be represented by patentometric analysis of the patent applicants, which are mainly companies. The 20 applicants who in total registered the most patents in this field from 2000 to 2018 are listed in Table 3.

**Table 3:** Number of patents registered by the top 20 applicants in the field of filamentous fungi from 2000 to 2018

Applicant	Number of patents
Novozymes	286
Danisco	179
DSM	140
Genencor	120
Toray Industries	75
Glykos Finland	66
Mark Aaron Emalfarb	46
Novartis	40
Kao	36
Marlow Foods	32
Toyo Boseki	32
VTT Technical Research Centre of Finland	30
IFP Énergies nouvelles	29
Michael Ward	29
Huaming Wang	29
Penn State Research Foundation	29
Meiji Seika	27
BASF	27
Institut national de la recherche agronomique	26
Elizabeth Bodie	24

Of the 2582 analysed patents, 1302 were registered by the top 20 applicants and 725 originated from the top four companies, Novozymes, Dansico, DSM and Genencor, combined. The list leader, Novozymes, alone registered 286 new patents in this period. However, 1280 patents were not registered by the top 20 applicants, showing that the range of industrial applicants is broad.

**Fig. 7:**
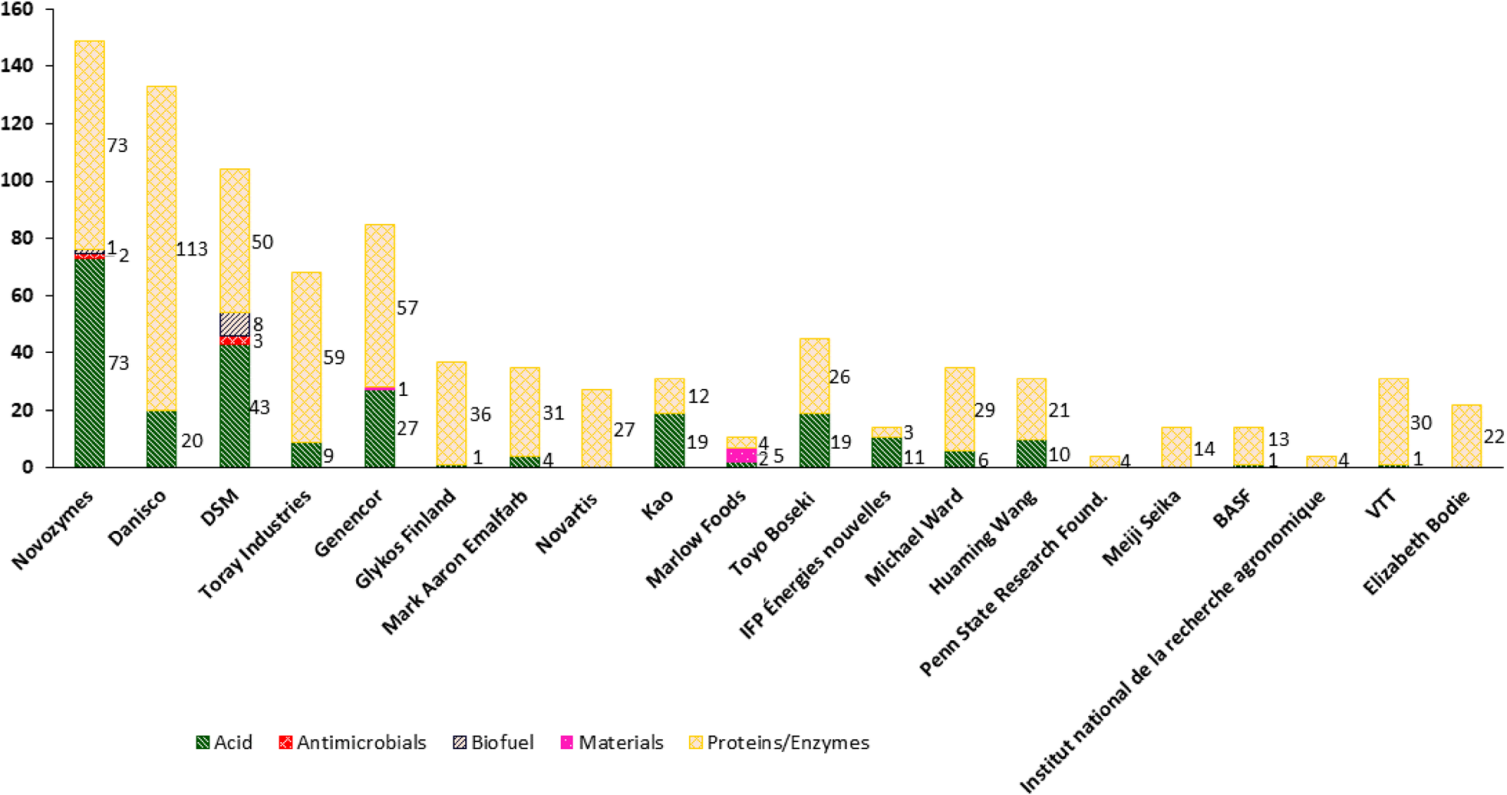
Products/applications of filamentous fungi by different applicants. The 20 applicants with the most patents from 2000 to 2018 are shown

To understand which products the top 20 companies focused on when applying filamentous fungi, patents were grouped into their uses/products for each company (Fig. 7). Note that not all patents could be assigned to a use/product.

Although most of the leading 20 companies predominantly used filamentous fungi for protein/enzyme production, acids were vital applications of these cell factories (e.g. Novozymes, Kao, Toyo Boseki and IFP Energies Nouvelles; Fig. 7). Notably, DSM was leading patent applications assigned as biofuel or antimicrobial related in this analysis, indicating a slightly more diverse portfolio of this company than others. No patents in the field of wastewater treatment or the production of antioxidants with filamentous fungal systems were found for the leading 20 companies from 2000 to 2018. Comparing the number of patents for the applications/products “wastewater”, “antioxidants”, “antimicrobials”, “biofuel” and “material” of all companies (Fig. 2B) with the leading 20 companies (Fig. 7) indicates that the patents for these applications/products are predominantly held by companies that are not part of this top 20 list.

## Discussion

Bibliometric and patentometric approaches enable quantitative analyses of research and intellectual property landscapes, respectively [12]. In contrast, review articles are always subjective to a certain extent and describe a field of research qualitatively. While there are several well-written and informative review articles about the usage and potential of filamentous fungi in biotechnology [7, 19], bibliometric approaches are missing. Hüttner et al. (2020) used a patentometric approach to quantitatively identify the key players of filamentous fungal biotechnology [11]. They also identified application areas (e.g. pharmaceuticals, bulk chemicals and enzymes) and highlighted trends (e.g. use of filamentous fungi as a food source or biodegradable materials, use in wastewater treatment and use in biorefineries). However, these fields were not analysed quantitatively. Elsewhere, Cerimi et al. (2019) used a patentometric approach to study bio-based materials, an upcoming subdiscipline in the exploitation of filamentous fungi [9]. In this study, we aimed to complement these previous works by combining bibliometric and patentometric approaches in a single study to give a comprehensive and quantitative assessment of primary filamentous fungal research and its translation into biotechnological applications.

From our analysis, a clear picture emerged whereby filamentous fungi are important drivers of biotechnological research and patent outputs. Further, the molecular and analytical tools available for filamentous fungi have improved considerably in recent years [7], at the same time implying great potential for increased application of filamentous fungi in biotechnology in the coming years. *Aspergillus* spp. were used most frequently, but *Trichoderma* and other genera were also key players. The fact that *Aspergillus* spp. were the first organisms applied in biotechnology (production of citric acid since more than 100 years) [6, 20] might be a possible explanation why they are in first place. Currently, enzyme and organic acid productions are the main applications, with novel technologies; for example, the use of biomass in construction is relatively less studied and with fewer patents. However, the EUROFUNG consortium proposes that filamentous fungi have the potential to play a major role to sustainably produce resilient sources of food, feed, chemicals, fuels, textiles, and materials in the future [7]. The ranking of products/applications of literature and patent outputs (Fig. 2) showed a similar behaviour. These data provided quantitative evidence that primary research can result in applied technological breakthroughs. However, the annual publication number of literature increased faster compared to patents (Fig. 1).

From a methodological perspective, the quality of data mined from the databases strongly influences the results of bibliometric and patentometric approaches. Thus, established databases, WoS and DEPATISnet, were used for bibliometrics and patentometrics, respectively. However, incomplete data sets can occur and influence the results [21]. Besides the database used, the search query is crucial. On the one hand, too broad search queries would result in many inappropriate publications/patents. On the other hand, many appropriate publications/patents will be missed when applying too narrow search queries. Thus, patentometric/bibliometric approaches always need to find a balance between the specificity of search queries and the resulting amount of output data. Based on this data analysis, research articles and patents can be assigned to more than one category. For example, enzymes used in biofuel processing may count into two categories: biofuel and enzymes. However, this possible limitation also offers opportunities; in future studies, interrelationships between different categories can be derived and explored in more detail.

This study applied bibliometric indices that rank the output of countries based on the number of citations. However, it must be mentioned that such indices should be evaluated with caution, especially because citations do not necessarily correlate with the quality of manuscripts [22, 23]. According to Haustein and Larivière (2015), it is reasonable to use more than one indicator [24]. Thus, we used the h-, g- and i10-indices, although we recognise that no group of metrics could perfectly encapsulate or summarise the value of a research field or subdiscipline.

## Conclusions

In this work we conducted quantitative analysis of literature (bibliometry) and patent (patentometry) data repositories to generate a snapshot of the state of filamentous fungal science in biotechnology. Five key trends were delineated from these datasets. This study demonstrated that filamentous fungi are important drivers of biotechnology. Enzyme and organic acid production by large companies remain the mainstay of filamentous fungal applications. Contrary, patents of less frequently used products/applications (“wastewater”, “antioxidants”, “antimicrobials”, “biofuel” and “material”) are predominantly held by companies that have few patents about filamentous fungi. Interestingly, *Aspergillus* was the most commonly used genus, but several other genera were widely employed by biotechnologists. Further, there was a broad range of companies that registered patents concerning filamentous fungal applications. China, the USA, Brazil and European countries (Germany, France, Spain, the Netherlands and England) emerged as key players concerning the total number of published literature. The evaluation of the quality of the published literature indicates the USA and the mentioned European countries as leaders in filamentous fungal science. However, the research community was global and highly interconnected by collaborations and citations. Thus, filamentous fungal science is global in nature and vital for biotechnology.

## Methods

Bibliometric and patentometric analyses were carried out from 2000 to 2020 and from 2000 to 2018, respectively. As patents get usually published (publication date marks the day a patent gets available to public) approximately 18 months after the filling date (day a patent gets registered), published patents were only investigated until a filling date at the end of 2018. The databases used, search queries applied and data analysis procedures are described as follows.

### Databases

Web of Science and DEPATISnet were chosen as databases for papers and patents, respectively. As one of the leading scientific databases, WoS has a high data coverage [25] and is often used for bibliometric analysis in different research fields [26–30]. DEPATISnet is one of the most important multinational patent databases available to the public free of charge and contains several million international patents [31]. Both WoS [25, 28] and DEPATISnet [31] enable the formulation of complex search queries and the export of their results for further analysis. Moreover, the well-presented user interfaces and exportation properties of WoS and DEPATISnet were factors to choose them over other databases.

### Search queries

Table 4 shows all search queries used in this study. To focus on product-oriented papers, “produ*” and “material*” were added. This was not necessary for patents as they already meet this requirement. Further, specified search queries concerning products and genera were applied by adding further search terms. To identify papers with specific search terms as the main content, these terms were only searched in a few field tags, namely title, author keywords and keywords plus. As patent databases provide less search options, the field tags for patents were chosen differently; more precisely, specific terms were only searched in the title and abstract.

**Table 4:** Applied search queries in Web of Science and DEPATISnet

Topics	Web of Science	Depatisnet
Filamentous fungi	TS = (filamentous-fung* AND (produ* OR material*)) AND PY = 2000–2020	(TI = (filamentous(W)fung?) OR AB = (filamentous(W)fung?)) AND (AY > = 2000 AND AY < = 2018)
Yeast	TS = (yeast* AND (produ* OR material*)) AND PY = 2000–2020	(TI = (yeast?) OR AB = (yeast?)) AND (AY > = 2000 AND AY < = 2018)
Fungi	TS = (fung* AND (produ* OR material*)) AND PY = 2000–2020	(TI = (fung?) OR AB = (fung?)) AND (AY > = 2000 AND AY < = 2018)
Bacteria	TS = (bacteri* AND (produ* OR material*)) AND PY = 2000–2020	(TI = (bacteri?) OR AB = (bacteri?)) AND (AY > = 2000 AND AY < = 2018)
Protozoa	TS = (protozo* AND (produ* OR material*)) AND PY = 2000–2020	(TI = (protozo?) OR AB = (protozo?)) AND (AY > = 2000 AND AY < = 2018)
Viruses	TS = ((virus* OR viral*) AND (produ* OR material*)) AND PY = 2000–2020	(TI = (virus? OR viral?) OR AB = (virus? OR viral?)) AND (AY > = 2000 AND AY < = 2018)
Plants	TS = (plant* AND (produ* OR material*)) AND PY = 2000–2020	(TI = (plant?) OR AB = (plant?)) AND (AY > = 2000 AND AY < = 2018)
Acid	TS = (filamentous-fung* AND (produ* OR material*)) AND (AK = (acid* OR malat* OR malic-acid* OR gluconat* OR gluconic-acid* OR itaconat* OR itaconic-acid* OR succinat* OR succinic-acid* OR citrat* OR citric-acid* OR fumarat* OR fumaric-acid*) OR KP = (acid* OR malat* OR malic-acid* OR gluconat* OR gluconic-acid* OR itaconat* OR itaconic-acid* OR succinat* OR succinic-acid* OR citrat* OR citric-acid* OR fumarat* OR fumaric-acid*) OR TI = (acid* OR malat* OR malic-acid* OR gluconat* OR gluconic-acid* OR itaconat* OR itaconic-acid* OR succinat* OR succinic-acid* OR citrat* OR citric-acid* OR fumarat* OR fumaric-acid*)) NOT (AK = (aminoacid* OR amino-acid*) OR KP = (aminoacid* OR amino-acid*) OR TI = (aminoacid* OR amino-acid*)) AND PY = 2000–2020	(TI = (filamentous(W)fung?) OR AB = (filamentous(W)fung?)) AND (TI = (acid? OR malat? OR malic#acid? OR gluconat? OR gluconic#acid? OR itaconat? OR itaconic#acid? OR succinat? OR succinic#acid? OR citrat? OR citric#acid? OR fumarat? OR fumaric#acid?) OR AB = (acid? OR malat? OR malic#acid? OR gluconat? OR gluconic#acid? OR itaconat? OR itaconic#acid? OR succinat? OR succinic#acid? OR citrat? OR citric#acid? OR fumarat? OR fumaric#acid?)) NOT (TI = amino#acid? OR AB = amino#acid?) AND (AY > = 2000 AND AY < = 2018)
Antimicrobials	TS = (filamentous-fung* AND (produ* OR material*)) AND (AK = (antifung* OR anti-fung* OR antimicrobi* OR anti-microbi* OR antibacteri* OR anti-bacteri* OR antibiotic* OR anti-biotic* OR antimycot* OR anti-mycot* OR antivir* OR anti-vir*) OR KP = (antifung* OR anti-fung* OR antimicrobi* OR anti-microbi* OR antibacteri* OR anti-bacteri* OR antibiotic* OR anti-biotic* OR antimycot* OR anti-mycot* OR antivir* OR anti-vir*) OR TI = (antifung* OR anti-fung* OR antimicrobi* OR anti-microbi* OR antibacteri* OR anti-bacteri* OR antibiotic* OR anti-biotic* OR antimycot* OR anti-mycot* OR antivir* OR anti-vir*)) AND PY = 2000–2020	(TI = (filamentous(W)fung?) OR AB = (filamentous(W)fung?)) AND (TI = (anti#fung? OR anti#microbi? OR anti#bacteri? OR anti#biotic? OR anti#mycot? OR anti#vir?) OR AB = (anti#fung? OR anti#microbi? OR anti#bacteri? OR antibiotic? OR anti#mycot? OR anti#vir?)) AND (AY > = 2000 AND AY < = 2018)
Antioxidants	TS = (filamentous-fung* AND (produ* OR material*)) AND (AK = (antioxidant* OR anti-oxidant*) OR KP = (antioxidant* OR anti-oxidant*) OR TI = (antioxidant* OR anti-oxidant*)) AND PY = 2000–2020	(TI = (filamentous(W)fung?) OR AB = (filamentous(W)fung?)) AND ((TI = anti#oxidant? OR AB = anti#oxidant?)) AND (AY > = 2000 AND AY < = 2018)
Biofuel	TS = (filamentous-fung* AND (produ* OR material*)) AND (AK = (ethanol* OR bioethanol* OR bio-ethanol* OR fuel* OR biofuel* OR bio-fuel* OR diesel* OR biodiesel* OR bio-diesel*) OR KP = (ethanol* OR bioethanol* OR bio-ethanol* OR fuel* OR biofuel* OR bio-fuel* OR diesel* OR biodiesel* OR bio-diesel*) OR TI = (ethanol* OR bioethanol* OR bio-ethanol* OR fuel* OR biofuel* OR bio-fuel* OR diesel* OR biodiesel* OR bio-diesel*)) AND PY = 2000–2020	(TI = (filamentous(W)fung?) OR AB = (filamentous(W)fung?)) AND (TI = (ethanol? OR bio#ethanol? OR fuel? OR bio#fuel? OR diesel? OR bio#diesel?) OR AB = (ethanol? OR bio#ethanol? OR fuel? OR bio#fuel? OR diesel? OR bio#diesel?)) AND (AY > = 2000 AND AY < = 2018)
Proteins/enzymes	TS = (filamentous-fung* AND (produ* OR material*)) AND (AK = (enzym* OR protein* OR peptid*) OR KP = (enzym* OR protein* OR peptid*) OR TI = (enzym* OR protein* OR peptid*)) AND PY = 2000–2020	(TI = (filamentous(W)fung?) OR AB = (filamentous(W)fung?)) AND (TI = (enzym? OR protein? OR peptid?) OR AB = (enzym? OR protein? OR peptid?)) AND (AY > = 2000 AND AY < = 2018)
Wastewater	TS = (filamentous-fung* AND (produ* OR material*)) AND (AK = (wastewater* OR waste-water* OR water-treatment*OR wastewater-treatment OR waste-water-treatment) OR KP = (wastewater* OR waste-water* OR water-treatment* OR wastewater-treatment OR waste-water-treatment) OR TI = (wastewater* OR waste-water* OR water-treatment* OR wastewater-treatment OR waste-water-treatment)) AND PY = 2000–2020	(TI = (filamentous(W)fung?) OR AB = (filamentous(W)fung?)) AND (TI = (waste#water? OR water#treatment? OR waste#water#treatment?) OR AB = (waste#water? OR water#treatment? OR waste#water#treatment?)) AND (AY > = 2000 AND AY < = 2018)
Materials	TS = (filamentous-fung* AND (produ* OR material*)) AND (AK = (composite* OR paper* OR leather* OR textil* OR packag* OR furniture*) OR KP = (composite* OR paper* OR leather* OR textil* OR packag* OR furniture*) OR TI = (composite* OR paper* OR leather* OR textil* OR packag* OR furniture*)) AND PY = 2000–2020	(TI = (filamentous(W)fung?) OR AB = (filamentous(W)fung?)) AND (TI = (composite? OR paper? OR leather? OR textil? OR packag? OR furniture?) OR AB = (composite? OR paper? OR leather? OR textil? OR packag? OR furniture?)) AND (AY > = 2000 AND AY < = 2018)
Aspergillus	TS = (filamentous-fung* AND (produ* OR material*)) AND (AK = aspergillus OR KP = aspergillus OR TI = aspergillus) AND PY = 2000–2020	(TI = (filamentous(W)fung?) OR AB = (filamentous(W)fung?)) AND ((TI = aspergillus? OR AB = aspergillus?)) AND (AY > = 2000 AND AY < = 2018)
Fusarium	TS = (filamentous-fung* AND (produ* OR material*)) AND (AK = fusarium OR KP = fusarium OR TI = fusarium) AND PY = 2000–2020	(TI = (filamentous(W)fung?) OR AB = (filamentous(W)fung?)) AND ((TI = fusarium? OR AB = fusarium?)) AND (AY > = 2000 AND AY < = 2018)
Neurospora	TS = (filamentous-fung* AND (produ* OR material*)) AND (AK = neurospora OR KP = neurospora OR TI = neurospora) AND PY = 2000–2020	(TI = (filamentous(W)fung?) OR AB = (filamentous(W)fung?)) AND ((TI = neurospora? OR AB = neurospora?)) AND (AY > = 2000 AND AY < = 2018)
Penicillium	TS = (filamentous-fung* AND (produ* OR material*)) AND (AK = penicillium OR KP = penicillium OR TI = penicillium) AND PY = 2000–2020	(TI = (filamentous(W)fung?) OR AB = (filamentous(W)fung?)) AND ((TI = penicillium? OR AB = penicillium?)) AND (AY > = 2000 AND AY < = 2018)
Trichoderma	TS = (filamentous-fung* AND (produ* OR material*)) AND (AK = trichoderma OR KP = trichoderma OR TI = trichoderma) AND PY = 2000–2020	(TI = (filamentous(W)fung?) OR AB = (filamentous(W)fung?)) AND ((TI = trichoderma? OR AB = trichoderma?)) AND (AY > = 2000 AND AY < = 2018)

Search field tags in WoS: TS: Title, Abstract, Authors Keywords, Keywords Plus; TI: Title; AK: Authors Keywords; KP: Keywords Plus; * Any group of characters or no character; xxx-yyy: “xxx-yyy” and “xxx yyy” (e.g. amino-acid and amino acid). Search field tags in DEPATISnet: TI: Title; AB: Abstract; AY: Application Year; ?: Any number of characters or no characters; #: One or no characters; (W): space

As the search query directly influences the resulting data, its definition is a key in bibliometric and patentometric analyses. To validate the chosen search queries, the coverage was defined as the ratio of publications that included at least one of the search words. For example, 59% of the investigated papers contained at least one of the following genera: *Aspergillus*, *Fusarium*, *Penicillium*, *Neurospora* and *Trichoderma* (Fig. 3).

### Export of data

For a detailed description how to export data from Web of Science and DEPATISnet, refer to Additional file 1: Supplementary Protocols 1 and 2, respectively. In general, both databases enable the export of results of search queries, including the contents of papers and patents, such as the title, abstract, and year of publication, in Excel tables and other formats, such as plain text files.

### Data analyses

Search results from Web of Science and DEPATISnet were analysed with Excel (version 2104; Microsoft), MATLAB (version 2020a; MathWorks), and VOSviewer (version 1.6.15; CWTS). While the data analysis procedures are described in detail in Additional file 1: Supplementary Protocols 1 and 2, the basic procedure is described in the following.

To compare the development of all publications/patents in Web of Science and DEPATISnet to publications/patents in the fields of filamentous fungi, fungi, bacteria, protozoa, viruses and plants, data were directly taken from WoS and DEPATISnet, respectively (Fig. 1; Table 1). Figures 2, 3 and 5 show the publication frequencies concerning filamentous fungi. For this purpose, the search results were exported to Excel and sorted and counted according to the publication year. To analyse the countries of origin of papers, the addresses of the corresponding authors were used. Author-networks (Fig. 6) were visualised with VOSviewer [16], a freely available software that enables the use of plain text files as input data. The bibliometric indices of each country (Table 2) were analysed in Excel, where it was differentiated amongst h-index [17], i10-index (Google Scholar), and g-index [18]. To determine genera-topic combinations (Fig. 4), search queries for genera and topics were combined, and the results were exported to and analysed in Excel. The most important applicants of patents (Table 3) were analysed semiautomatically with MATLAB and Excel. Knowing the most important applicants, their occurrence in different topics was counted in Excel (Fig. 7).

## Supplementary Information

**Additional file 1.** Protocols for bibliometric and patentometric analyses.

## Data Availability

The datasets used and/or analysed in this study are available from the corresponding author on reasonable request.

## Abbreviations

WoS: Web of Science
spp.: Species
*A*.: *Aspergillus*
CAZys: Carbohydrate activating enzymes
