# Supplementary material for: Filamentous fungal applications in biotechnology: a combined bibliometric and patentometric assessment
Source: Fungal Biol Biotechnol. 2021 Dec 28;8:23. doi: 10.1186/s40694-021-00131-6 (PMC8713403; doi:10.1186/s40694-021-00131-6)
Supplement: Supplementary file 1 — Additional file 1. Protocols for bibliometric and patentometric analyses. [file 40694_2021_131_MOESM1_ESM.docx]

**Additional file 1.**

**Protocol 1: Bibliometry**

*1. Advanced Search in Web of Science was selected.*

*2. Search query*

A search query similar to Table 4 was selected, e.g. TS = (bacteri* AND (produ* OR material*)) AND PY = 2000-2020

*3. Export of data*

- Open *Results* of search query.
- Select all results and *Add to Market List*.
- Save/export results from *Market List*.
- *Select records*: Only the information of 500 papers can be downloaded at once. If results contained >500 papers, they were downloaded sequentially, i.e. first papers 1 to 500, second papers 501 to 1000, …
- *Select content* that should be exported for each paper, e.g. authors, title, keywords and abstract. Select *All*.
- *Select destination*: File format can be chosen. Results were exported in *.xls files (Excel) and plain text files.
- If there were > 500 papers, the search was split (see above), and the files were merged afterwards.
- Each line of exported Excel tables contains one paper, and columns show the content of each paper.

*4. Data analysis*

- Figure 1/Table 1
- Step 1: A search query was selected, e.g. TS = (bacteri* AND (produ* OR material*)) AND PY = 2000-2020
- Step 2: In Web of Science, under *Refine Results*, the number of publications was read off per year.
- Step 3: Steps 1 and 2 were repeated for fungi, filamentous fungi, protozoa, viruses and plants.
- Step 4: To identify the total number of publications in WoS, the search query PY = (2000-2020) was used.
- Figure 2
- Step 1: A search query was selected, e.g. TS = (filamentous-fung* AND (produ* OR material*)) AND (AK = (enzym* OR protein* OR peptid*) OR KP = (enzym* OR protein* OR peptid*) OR TI = (enzym* OR protein* OR peptid*)) AND PY = 2000-2020
- Step 2: Results were exported to Excel.
- Step 3: With the help of a pivot table in Excel, the publications were sorted and counted according to their publication year.
- Step 4: Steps 1 to 3 were repeated for acid, antimicrobials, antioxidants, biofuel, materials and wastewater.
- Figure 3
- Step 1: A search query was selected, e.g. TS = (filamentous-fung* AND (produ* OR material*)) AND (AK = aspergillus OR KP = aspergillus OR TI = aspergillus) AND PY = 2000-2020
- Step 2: Results were exported to Excel.
- Step 3: With the help of a pivot table in Excel, the publications were sorted and counted according to their publication year.
- Step 4: Steps 1 to 3 were repeated for *Fusarium*, *Neurospora*, *Penicillium* and *Trichoderma*.
- Figure 4
- Step 1: A search query combining one genera (e.g. *Aspergillus*) and one topic (e.g. proteins) was selected, e.g. TS = (filamentous-fung* AND (produ* OR material*)) AND (AK = aspergillus OR KP = aspergillus OR TI = aspergillus) AND .(AK = (enzym* OR protein* OR peptid*) OR KP = (enzym* OR protein* OR peptid*) OR TI = (enzym* OR protein* OR peptid*)) AND PY = 2000-2020
- Step 2: Results were exported to Excel, and the number of publications was counted.
- Step 3: Steps 1 and 2 were repeated for all combinations of genera and topics.
- Figure 5
- Step 1: Search query TS = (filamentous-fung* AND (produ* OR material*)) AND PY = 2000-2020 was selected.
- Step 2: Search results were filtered by country in WoS.
- Step 3: Results of the 10 countries with the most publications were exported separately in Excel.
- Step 4: With the help of a pivot table in Excel, the publications of each country were sorted and counted according to their publication year.
- Figure 6
- Step 1: Search query TS = (filamentous-fung* AND (produ* OR material*)) AND PY = 2000-2020 was selected.
- Step 2: Results were exported as plain text files.
- Step 3: Map based on bibliographic data was created in VOSviewer with data from bibliographic database file (see above; exported as plain text files)
- Step 4: To visualise the coauthorship network, the following parameters were selected: countries, full counting, and number of countries to be selected: 20.
- Step 5: To visualise the citation network, the following parameters were selected: countries and number of countries to be selected: 20.
- Table 2
- Step 1: Search query TS = (filamentous-fung* AND (produ* OR material*)) AND PY = 2000-2020 was selected.
- Step 2: Search results were filtered by country in WoS.
- Step 3: Filtered results of each country were exported separately to Excel.
- Step 4: The papers of each country were ranked and numbered in Excel based on their number of citations.
- Step 5: The h-, i10- and g-indices of each country were analysed based on the ranked paper.

**Supplementary Protocol 2: Patentometry**

*1. Expert Search in* DEPATISnet *was selected.*

*2. Search query*

A search query similar to Table 4 was selected, e.g. (TI = (filamentous(W)fung?) OR AB = (filamentous(W)fung?)) AND (AY >= 2000 AND AY <= 2018)

*3. Export data*

- DEPATISnet can only display 10,000 patents at once. If the results contained >10,000 patents, they were divided into shorter periods, e.g. 2000–2004, 2005–2009, …
- *Result list configuration*: Select content that should be exported for each patent, e.g. title abstract, inventor and applicant. Select *All*.
- Select a file format (next to *download*). csv-files were used.
- If the results had to be split into several periods (see above), csv-tables (compatible with Excel) were merged afterwards.

*4. Data analysis*

- Figure 1/Table 1
- Step 1: A search query was selected, e.g. (TI = (bacteri?) OR AB = (bacteri?)) AND (AY >= 2000 AND AY <= 2018)
- Step 2: The number of patents was read off per year.
- Step 3: Steps 1 and 2 were repeated for fungi, filamentous fungi, protozoa, viruses and plants.
- Step 4: To identify the total number of patents in DEPATISnet, the basic search was applied for certain time ranges without any other specifications.
- Figure 2
- Step 1: A search query was selected, e.g. (TI = (filamentous(W)fung?) OR AB = (filamentous(W)fung?)) AND (TI = (enzym? OR protein? OR peptid?) OR AB = (enzym? OR protein? OR peptid?)) AND (AY >= 2000 AND AY <= 2018)
- Step 2: Results were exported to Excel.
- Step 3: With the help of a pivot table in Excel, the publications were sorted and counted according to their publication year.
- Step 4: Steps 1 to 3 were repeated for acid, antimicrobials, antioxidants, biofuel, materials and wastewater.
- Figure 3
- Step 1: A search query was selected, e.g. (TI = (filamentous(W)fung?) OR AB = (filamentous(W)fung?)) AND ((TI = aspergillus? OR AB = aspergillus?)) AND (AY >= 2000 AND AY <= 2018)
- Step 2: Results were exported to Excel.
- Step 3: With the help of a pivot table in Excel, the publications were sorted and counted according to their publication year.
- Step 4: Steps 1 to 3 were repeated for *Fusarium*, *Neurospora*, *Penicillium* and *Trichoderma*.
- Figure 7
- Step 1: A search query concerning a product group was selected, e.g. (TI = (filamentous(W)fung?) OR AB = (filamentous(W)fung?)) AND (TI = (enzym? OR protein? OR peptid?) OR AB = (enzym? OR protein? OR peptid?)) AND (AY >= 2000 AND AY <= 2018)
- Step 2: Results were exported to Excel.
- Step 3: The patents of the most important applicants were counted manually based on the Excel table.
- Step 4: Steps 1 to 3 were repeated for acid, antimicrobials, antioxidants, biofuel, materials and wastewater
- Table 3
- Step 1: A search query was selected, e.g. (TI = (filamentous(W)fung?) OR AB = (filamentous(W)fung?)) AND (AY >= 2000 AND AY <= 2018)
- Step 2: Results were exported to Excel.
- Step 3: Applicants were sorted and counted with a self-written code in MATLAB; this list of applicants was exported to Excel.
- Step 4: Due to different spellings of applicants, they were finally checked and assigned manually in Excel.
